# Effect of quantitative versus qualitative neuromuscular blockade monitoring on rocuronium consumption in patients undergoing abdominal and gynecological surgery: a retrospective cohort study

**DOI:** 10.1007/s10877-022-00909-y

**Published:** 2022-08-20

**Authors:** Lea Valeska Blum, Ellen Steeger, Sonja Iken, Gösta Lotz, Sebastian Zinn, Florian Piekarski, Kai Zacharowski, Florian Jürgen Raimann

**Affiliations:** Department of Anesthesiology, Intensive Care Medicine and Pain Therapy, University Hospital Frankfurt, Goethe University, Theodor-Stern-Kai 7, 60590 Frankfurt, Germany

**Keywords:** Train of four, Qualitative monitoring, Quantitative monitoring, Neuromuscular blocker, Continuous relaxation

## Abstract

The level of neuromuscular blockade can be assessed by subjective (qualitative) and objective (quantitative) methods. This study aims to compare the dosage of the neuromuscular blocking agents (NMBA) rocuronium and the need for reversion by sugammadex between those methods. A retrospective, observational analysis was conducted. In the tactile qualitative-neuromuscular monitoring-group (tactile NMM) (n = 244), muscle contractions were assessed tactilely. In the quantitative neuromuscular monitoring-group (n = 295), contractions were accessed using an acceleromyograph. Primary endpoints were dosage of rocuronium per minute operation-time (milligram per kilogram bodyweight per minute (mg/kgBW/min)), count of repeated rocuronium administrations and use of sugammadex. Secondary endpoints were: NMM use before repeated NMBA application or extubation, time to extubation, post-operative oxygen demand. A total of n = 539 patients were included. n = 244 patients were examined with tactile NMM and 295 patients by quantitative NMM. Quantitative NMM use resulted in significantly lower rocuronium dosing (tactile NMM: 0.01 (± 0.007) mg/kgBW/min vs. quantitative NMM: 0.008 (± 0.006) mg/kgBW/min (*p* < 0.001)). In quantitative NMM use fewer repetitions of rocuronium application were necessary (tactile NMM: 83% (n = 202) vs. quantitative NMM: 71% (n = 208) *p* = 0.007). Overall, 24% (n = 58) in the tactile NMM-group, and 20% (n = 60) in the quantitative NMM-group received sugammadex ((*p* = 0.3), OR: 1.21 (0.81–1.82)). Significantly fewer patients in the quantitative NMM-group required oxygen-supply postoperative (quantitative NMM: 43% (n = 120)) vs. tactile NMM: 57% (n = 128)) (*p* = 0.002). The use of quantitative assessment of NMBA results in a lower overall dosage and requires fewer repetitions of rocuronium application. Therefore, quantitative monitoring systems should be used to monitor NMBA intraoperatively to reduce NMBA dosing, while achieving continuous neuromuscular blockade.

## Introduction

Anaesthetized patients are often treated with neuromuscular blocking agents (NMBA) [1]. Especially in laparoscopic surgery a deep muscular blockade may even provide better surgical conditions [2–4].

Disadvantages of any neuromuscular block are in case of incomplete residual neuromuscular blockade complications, such as aspiration, postoperative hypoxemia, pneumonia, and atelectasis [5–9]. Thus, neuromuscular monitoring can be used for quantification to reduce the likelihood of residual neuromuscular blockade [10].

Neuromuscular monitoring is commonly based on the train-of-four (TOF) stimulation of the ulnar nerve [10]: a series of four supramaximal pulses are applied, the degree of the neuromuscular blockade is measured with the decrement of response of the adductor pollicis muscle to repetitive nerve stimulation. Amplitude or decrement of responses can be quantified either electromyographically or acceleromyographically or estimated with tactile sense [11]. In conventional, tactile neuromuscular monitoring (tactile NMM), muscle contractions are assessed tactilely by the examiner. As a result, this method is prone to sources of error such as changing examiners and different empirical values in the assessment of contraction and thus can be misinterpreted [12].

The differences between intraoperative acceleromyography and tactile NMM analysis by the examiner have already been investigated with regard to postoperative complications resulting from residual blockade [13].

The present study aimed to compare the dosage of the muscle relaxant rocuronium and use of sugammadex between qualitative, tactile neuromuscular monitoring and quantitative neuromuscular monitoring, to proof our hypothesis, that quantitative e monitoring can be used to control the dosage of NMBAs more precisely and therefore contribute a stable level of relaxation than with tactile monitoring.

## Methods

Approval for the present study was obtained from the local ethics committee of the University Hospital Frankfurt (No. 20-1065) and the study was performed in accordance with the actual Declaration of Helsinki. All data were anonymized. Due to its retrospective design the study was not registered in a trial database.

### Study design

**Fig. 1:**
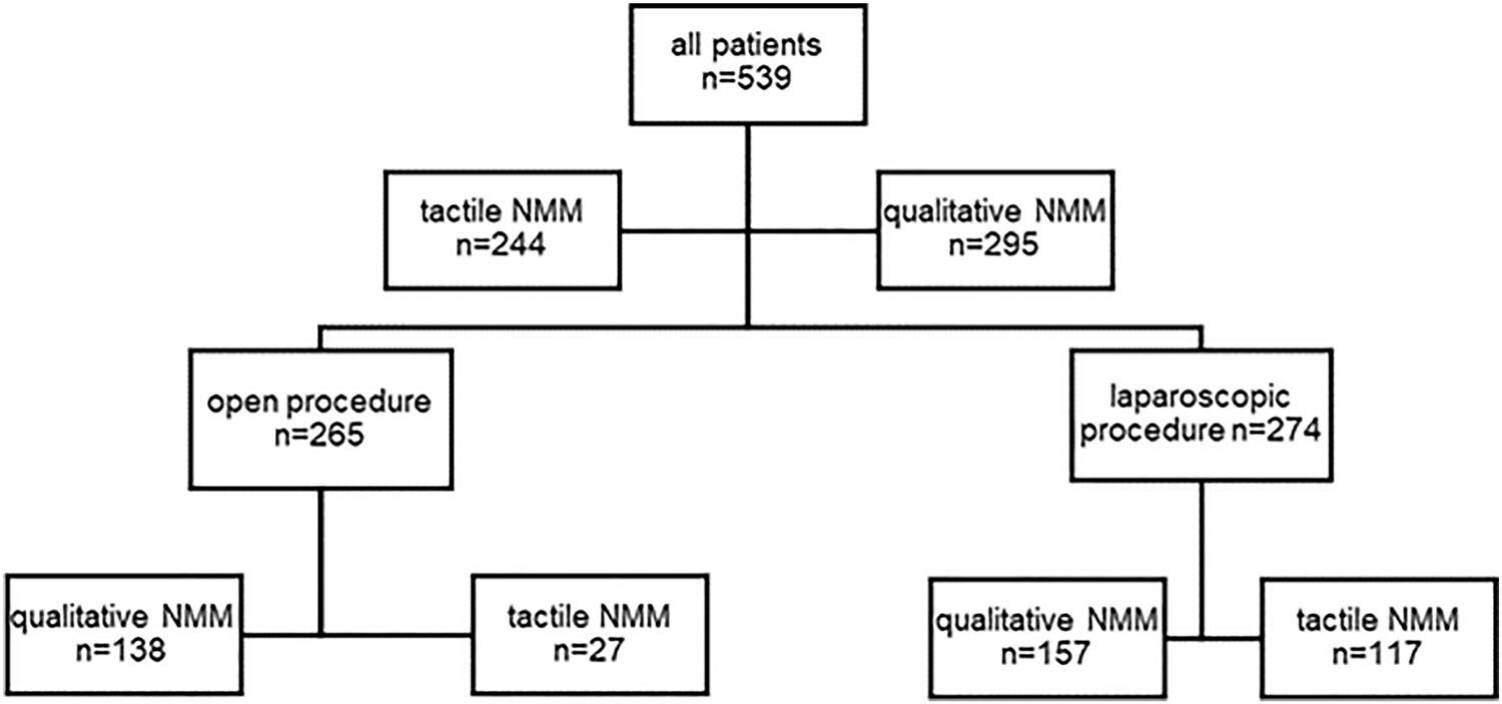
Study Groups

For this retrospective observational analysis, patients were divided into two groups: a tactile NMM-group and a quantitative NMM-group. As a sub-analysis, we examined patients undergoing open abdominal and gynecological surgery and patients undergoing laparoscopic surgery separately (Fig. 1). The analyzed operations took place between August 2018 and December 2020. For general and abdominal surgery tactile neuromuscular monitoring was the only monitoring available from August 2018 until August 2019, and for gynecology from December 2018 until December 2019, respectively. The tactile NMM was replaced with qualitative neuromuscular monitoring in August 2019 and December 2019, respectively. Patients older than 18 years of age, in whom one of the aforementioned TOF techniques was used and who received rocuronium as an NMBA were included. If reversion was necessary, it was performed with sugammadex. In both groups propofol was used for anesthesia induction, while maintenance of anesthesia was achieved by volatile anesthetics. Patients younger than 18 years of age, requiring rapid sequence induction, suffering from liver dysfunction, and preexisting neuromuscular disorder were excluded. As neuromuscular monitoring was not used in every surgery, patients without documented neuromuscular monitoring were excluded. Primary endpoints were dosage of rocuronium (Esmeron^®^ Inresa, Freiburg, Germany) per minute of operation time (milligram per kilogram bodyweight per minute (mg/kgBW/min), count of repeated rocuronium applications and use of sugammadex (Bridion^®^ Merck Sharp & Dohme B.V., Harleem, Netherlands). Secondary endpoints were: Use of neuromuscular monitoring before repeated application of NMBA or extubation, time between end of operation and extubation as well as post-operative oxygen demand.

Tactile qualitative neuromuscular monitoring was used in the tactile NMM-group (Device: NS 252 Fisher & Paykel Healthcare Limited, Auckland, New Zealand), while in the quantitative NMM-group, quantitative monitoring with an acceleration transducer was used (TOFScan^®^, Dräger, Lübeck, Germany) to monitor neuromuscular blockade. The investigations were carried out as: four supramaximal pulses of 0.2 ms duration, with a frequency of 2 Hz. Free movement of the thumb was ensured in all evaluated patients.

The operation schedule was searched for abdominal and gynecological surgeries (open and laparoscopic surgery). The required data was extracted from digital anesthesiologic protocols (Sandman.MD © App@work, Berlin, Germany) and post-anesthesia care unit (PACU) protocols (Sandman.MD © App@work, Berlin, Germany). The collected data included dosage of rocuronium and use of sugammadex, (if applied), basic demographic data, bodyweight, American Society of Anesthesiologists (ASA) Classification [14], type and duration of the surgical procedure (hours:minutes:seconds [hh:mm:ss]) and time between the end of operation and extubation (minutes:seconds [mm:ss]). In the PACU, a newly emerged oxygen demand was recorded.

### Statistical analysis

The data were analyzed using IBM SPSS Statistics (IBM Corp. Released 2020. IBM SPSS Statistics for Macintosh, Version 27.0. Armonk, NY: IBM Corp). Values are expressed as number (percentage), descriptive variables were calculated using mean with standard deviation (SD) (for normally distributed continuous data with homogeneous variance) or median with range (min–max). A *p* value < 0.05 was considered to be statistically significant. Depending on distribution (determined by the Shapiro–Wilk test), the Student’s t-test or Mann–Whitney-U test was used. The Kruskal–Wallis test was applied for group comparisons. The odds ratio (OR) was used to measure the association between exposure and outcome.

## Results

In total, n = 539 patients were analyzed. Tactile NMM was used to examine 244 patients, and 295 patients were examined with quantitative NMM (Fig. 1).

### Patients’ characteristics

We examined significantly more female patients (tactile NMM n = 139, quantitative NMM n = 201). Overall, 38% (n = 205) of the n = 539 operations were performed in the field of gynecology. All other cases were abdominal or general surgical procedures. There was no significant difference in mean age at surgery between the two groups: 56 ± 16.9 years in the tactile NMM-group and 53.2 ± 16.4 years in the quantitative NMM-group. Median ASA classification was comparable between the tactile NMM-group and the quantitative NMM-group (2 (1–4) vs. 2 (1–4), respectively). A regression analysis was performed to identify possible confounders for rocuronium dosage and found age (*p* = 0.004), initial rocuronium dosage (*p* < 0.001) and operating time (*p* < 0.001) to be significant. ASA-classification, gender and surgical technique (open vs. laparoscopic surgery) had no significant influence.

All identified confounders were included in a multivariate analysis (Table 1).

**Table 1:** Keyfactors (tactile and qualitative NMM) and possible cofactors for the primary endpoints

Cofactors	Keyfactors		Univariate effect size (R) with respect to the keyfactor
	Tactile NMM (n = 244)	Qualitative NMM (n = 295)	
Sex (female), count (%)	57 (n = 139)	68 (n = 201)	0.12
Gynecological cases, count (%)	30 (n = 72)	45 (= 133)	0.16
Age (years), mean ± SD	56 ± 16.9	53.2 ± 16.4	0.09
ASA median, range (min–max)	2 (1–4)	2 (1–4)	0.003
Surgical technique: open versus laparoscopic	Open: n = 127 Laparoscopic n = 117	Open: n = 138 Laparoscopic: n = 157	0.05
Initial rocuronium dosage (mg/kgBW), mean ± SD	0.57 ± 0.13	0.54 ± 0.13	0.12
Operating time (hh:mm:ss) ± SD	2:18:53 ± 1:38:45	2:09:38 ± 1:49:00	0.44

Results are depicted as mean (± SD (standard deviation)) or median (range), n = count (percentage), n.s. = not significant

*NMM* neuro-muscular monitoring

### Rocuronium-administration

**Fig. 2:**
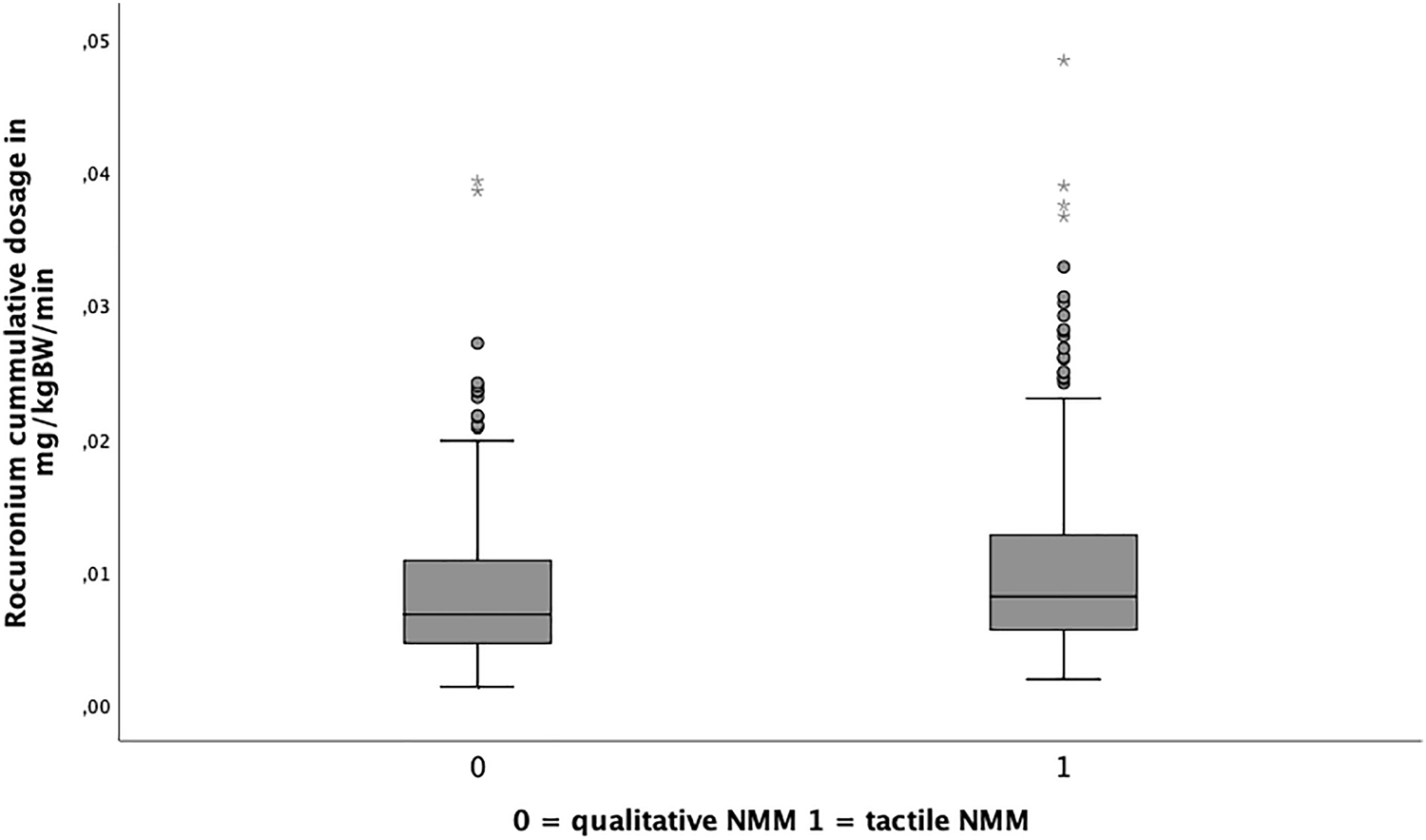
Boxplots, comparing dosage of rocuronium per minute surgery-time

We found a significant difference in dosage of rocuronium per minute operation time (tactile NMM: 0.01 (± 0.007) mg/kgBW/min vs. quantitative NMM 0.008 (± 0.006) mg/kgBW/min (*p* < 0.001)) (Fig. 2).

A significant difference was also detected in the repetitive administration of the NMBA, which was necessary significantly more often if tactile NMM was used (83% (n = 202) vs. quantitative NMM 71% (n = 208; *p* = 0.007) OR 2.0 (1.3–3.1) (Table 2).

**Table 2:** Outcomes

	Tactile NMM (n = 244)	Qualitative NMM (n = 295)	*p* value	OR
Initial rocuronium dosage (mg/kgBW), mean ± SD	0.55 ± 0.13	0.52 ± 0.11	0.03	
Repetitive NMBA administrations, count (%)	83 (n = 202)	71 (n = 208)	0.007	2.0 (1.3–3.1)
Rocuronium dosage per minute operating-time (mg/kgBW/min), mean ± SD	0.01 (± 0.007)	0.008 (± 0.006)	< 0.001	
NMM use before repeated NMBA administration count (%)	25 (n = 62)	46 (n = 136)	< 0.001	0.4 (0.28–0.58)
NMM use before extubation count (%)	92% (n = 225)	94% (n = 276)	n.s.	0.82 (0.42–1.58)
Sugammadex-administration, count (%)	20 (n = 23)	21 (n = 33)	n.s.	0.92 (0.51–1.67)
Time to extubation (mm:ss), mean ± SD	10:00 ± 09:19	10:59 ± 13:17	n.s.	
Oxygen administration in PACU*, count (%)	57 (n = 128)	43 (n = 120)	n.s.	1.21 (0.81–1.83)
Oxygen administration in PACU in patients without Sugammadex-administration**, count (%)	53 (n = 91)	39 (n = 86)	0.006	1.75 (1.17–2.63)

Results are depicted as mean (± SD (standard deviation)) or median (range), n = count, n.s. = not significant

*NMDA* neuro-muscular blocking agents, *NMM* neuro-muscular monitoring

*Deviating number of patients n = 501,

** Deviating number of patients n = 209

In a multivariate analysis including the type of NMM, gender, surgical discipline and technique, initial rocuronium dosage and operating time the factors type of NMM, surgical discipline, initial rocuronium dosage and operating time showed a significant effect on the rocuronium dosage, as well as the repetitive NMBA administration (Table 3).

**Table 3:** Multivariate analysis of potential independent risk factors type of NMM, gender, surgical discipline, surgical technique, initial rocuronium dosage, operating time on primary outcomes, rocuronium dosage per minute operating-time, count of repetitive NMBA administration, sugammadex-administration and secondary outcomes NMM use before repeated NMBA application or extubation, time to extubation, post-operative oxygen demand

	Rocuronium dosage per minute operating-time	Mean count of repetitive NMBA administrations	Sugammadex-administration (odds-ratio)	NMM use before repeated NMBA administration (odds-ratio)	NMM use before extubation (odds-ratio)	Time to extubation	Oxygen administration in PACU* (odds-ratio)	Oxygen administration in PACU in patients without Sugammadex-administration**
Type of NMM (tactile)	*p* < 0.001	*p* = 0.01	n.s. (*p* = 0.79) OR: 0.95 (0.62–1.44)	*p* < 0.001 OR 2.71 (1.8–4.1)	n.s. (*p* = 0.52) OR: 1.25 (0.62–2.5)	n.s. (*p* = 0.14)	*p* = 0.02 OR: 0.58 (0.37–0.91)	*p* = 0.02 OR: 0.56 (0.33–0.93)
Gender (male)	*p* = 0.6	n.s. (*p* = 0.51)	n.s. (*p* = 0.08) OR: 1.57 (0.95–2.6)	n.s. (*p* = 0.6) OR: 0.88 (0.53–1.46)	n.s. (*p* = 0.82) OR: 1.06 (0.45–2.52)	n.s. (*p* = 0.06)	n.s. (*p* = 0.4) OR: 1.28 (0.76–2.15)	n.s. (*p* = 0.81) OR: 0.94 (0.51–1.71)
Surgical discipline (Gyn)	*p* < 0.01	*p* = 0.03	*p* = 0.006 OR: 2.13 (1.24–3.65)	n.s. (*p* = 0.7) OR: 0.95 (0.57–1.59)	n.s. (*p* = 0.93) OR: 0.95 (0.38–2.38)	*p* < 0.001	*p* < 0.001 OR: 14.6 (7.66–27.7)	*p* < 0.001 OR: 13.1 (6.2–27.69)
Surgical technique (open)	n.s. (*p* = 0.8)	n.s. (*p* = 0.2)	n.s. (*p* = 0.68) OR: 1.1 (0.69–1.78)	n.s. (*p* = 0.73) OR: 1.07 (0.68–1.67)	n.s. (*p* = 0.53) OR: 0.71 (0.32–1.6)	*p* = 0.04	*p* = 0.001 OR: 2.07 (1.3–3.3)	*p* = 0.001 OR: 2.49 (1.45–4.27)
Initial rocuronium dosage	*p* < 0.001	*p* = 0.04	*p* = 0.02 OR 6.96 (1.41–34.19)	n.s. (*p* = 0.72) OR: 0.67 (0.16–3.38)	n.s. (0.65) OR: 0.5 (0.04–6.35)	*p* = 0.006	n.s. (*p* = 0.78) OR: 0.71 (0.15–3.49)	n.s. (*p* = 0.66) OR: 0.62 (0.1–3.87)
Operating time	*p* < 0.001	*p* < 0.001	n.s. (*p* = 0.31)	*p* < 0.001	*p* = 0.002	n.s. (*p* = 0.166)	*p* < 0.001	*p* < 0.001

*PACU* post operative care unit, *NMM* neuro-muscularmonitoring, n.s = not significant

*Deviating number of patients n = 501

**Deviating number of patients n = 209

### TOF-usage

Patients in the quantitative NMM-group were tested significantly (*p* < 0.001) more often before repeated administration of NMBAs (tactile NMM: 25% (n = 62) vs. quantitative NMM: 46% (n = 136) OR 0.4 (0.28–0.58)). No significant difference could be detected between testing for rest-relaxation before extubating between both groups (tactile NMM: 92% (n = 225) vs. quantitative NMM: 94% (n = 276) (OR: 0.82 (0.42–1.58)) (Table 2). In a multivariate analysis including the type of NMM, gender, surgical discipline and technique, initial rocuronium dosage and operating time the factors type of NMM and operating time showed a significant effect on the NMM use before repeated NMBA administration. Operating time showed a significant effect on NMM use before extubation (Table 3).

### Sugammadex-usage

In the tactile NMM-group, 24% (n = 58), and 20% (n = 60) in the quantitative NMM-group received sugammadex (Table 2).

In a multivariate analysis including the type of NMM, gender, surgical discipline and technique, initial rocuronium dosage and operating time the factors surgical discipline (OR: 2.13 (1.24–3.65)) and initial rocuronium dosage (OR 6.96 (1.41–34.19)) showed a significant effect on the sugammadex-administraion (Table 3).

The time from suture completion to extubation did not differ significantly between both groups (tactile NMM: 10:00 ± 09:19 min vs. quantitative NMM 10:59 ± 13:17 min), as well as the overall surgical time (01:45:59 ± 1:38:45 vs. 01:40:00 ± 1:49:00)) (Table 2).

Patients from the tactile NMM-group required significant more oxygen application in the PACU when compared to those from the quantitative NMM-group (n = 128 (57%) vs. n = 120 (43%), respectively; OR 1.21 (0.81–1.83)). The number of analyzed patients (due to lack of PACU surveillance in some patients) in this analysis was n = 501. In Patients who did not receive sugammadex the tactile NMM-group required significant more oxygen application in the PACU as well (n = 91 (53%) vs. n = 86 (39%), respectively; OR 1.75 (1.17–2.63)) (Table 2).

In a multivariate analysis including the type of NMM, gender, surgical discipline and technique, initial rocuronium dosage and operating time the factors type of NMM, surgical discipline and technique and operating time showed a significant effect on the oxygen administration in PACU for all patients. Including only patients who did not receive sugammadex factors type of NMM, surgical discipline and technique and operating time showed a significant effect on the oxygen administration in PACU as well (Table 3).

### Subanalysis

In the subanalysis, n = 265 patients undergoing open abdominal surgery and n = 274 undergoing laparoscopic surgery were compared.

Within the open surgery group, n = 127 (47.9%) patients were allocated to the tactile NMM-group and n = 138 (52.1%) patients to the qualitative NMM-group. Within the laparoscopic surgery group, n = 117 (42.7%) patients were examined with tactile NMM and n = 157 (57.3%) with quantitative NMM. We found significant differences in open surgery for rocuronium dosage per minute operation time time with higher dosage in the tactile NMM-group (*p* < 0.001). No significant difference in neuromuscular monitoring use prior to repeated NMBA application was detected. No significant differences were detected in mean age, sugammadex administration (OR = 1.56 (0.88–2.77)).

In laparoscopic surgery the rocuronium dosage per minute operation time was significantly (*p* = 0.006) higher in patients in the tactile NMM-group. In the quantitative NMM-group, monitoring was used significantly more often before a NMBA application was repeated (*p* < 0.001).

## Discussion

The present study aimed to compare the dosing of rocuronium and sugammadex usage in regard of two different neuromuscular monitoring methods in 539 patients. We observed significantly higher cumulative doses of rocuronium in patients that were monitored with tactile NMM than patients who were treated by quantitative NMM. Nevertheless, overall sugammadex usage was comparable.

We were able to demonstrate, that in the quantitative NMM-group increments in NMBA-dosage were less frequently administered and the indication to give increments was more frequently guided by quantitative NMM. This could be explained by a better evaluation of the relaxation status of the patient via quantitative NMM, as well as increased user-friendliness and, derived from this, a more accurate dosage of NMBAs.

Recently, most studies in this field compare no monitoring versus tactile NMM, which is shown in the meta-analysis by Naguib et al., who analyzed 53 studies and showed a lower rate of residual curarisation in studies using intraoperative neuromuscular monitoring [13]. Only one of the analyzed studies distinguished between conventional monitoring and acceleromyography [15]; no sub-analysis dividing laparoscopic and open surgery was performed, thus our study is the first to evaluate the influence quantitative NMM in this context. In laparoscopic operations, the patient’s arms are sometimes attached to the patient’s flank, so the acceleromyograph must be attached to the patient’s hand before positioning. If neuromuscular blockade is monitored more frequently and evaluation is objectified via an acceleromyograph, a decrease in relaxation might be detected earlier. This could explain the lower overall dosage of NMBA, with significantly less incremental doses when quantitative NMM is used. The assessment of the count of repeat doses is subject to the bias of the operation time, which did not differ between groups in this study. It seems reasonable to conclude a stable level of neuromuscular blockade is more likely to be maintained in the quantitative NMM, whereas an “up and down” neuromuscular blockade using tactile NMM. Especially in laparoscopic surgery, the neuromuscular block is known to optimize space conditions, shown by a meta-analysis by Bruintjes et al. in 2017 [16]. This might explain our results in the subanalysis: we observed higher dosages of NMBA in laparoscopic surgery than in open surgery and, also higher repeated administrations differences in repetitive administration in the quantitative NMM. In a randomized-controlled clinical trial, Veelo et al. examined 69 patients undergoing thoraco-laparoscopic esophagectomy and compared continuous rocuronium infusion with on-demand neuromuscular blockade, both measured using quantitative NMM. The continuous dosage was 0.6 mg/kgBW/min. There was no improvement of surgical conditions, no difference in postoperative pain scores and no difference in duration of surgery between on-demand rocuronium boluses and continuous application [17]. The dosages measured in our study using the quantitative NMM were almost the same as the continuous rate in this study, even though they were given as boluses. Therefore, it seems reasonable to assume that a nearly continuous neuromuscular blockade can be achieved using the quantitative NMM.

We did not control the neuromuscular function in the PACU. However, the more frequent need for oxygen in the recovery room in patients with tactile NMM monitoring might be explained by a higher rate of unrecognized residual neuromuscular block. This is in accordance to Murphyet al., who compared 185 patients and showed a significantly reduced incidence, severity and duration of hypoxemic events in patients monitored by acceleromyographic monitoring [18]. This leads to the conclusion that a residual blockade is more likely to be misdiagnosed using the tactile NMM. Kotake et al. demonstrated that even with NMBA reversal via sugammadex, a risk of residual blockade exists and needs to be identified using neuromuscular monitoring [19]. In contrast, the POPULAR-study by Kirmeier et al. demonstrated in a multicenter, prospective observational cohort study that the use of NMBAs is associated with an increased risk of postoperative pulmonary complications, but the use of neuromuscular monitoring (both tactile and quantitative) was not associated with a reduction of this risk [20], while a secondary analysis showed that a raise of the TOF-ratio > 0.95 (instead of > 0.9) reduced the adjusted risk of postoperative pulmonary complications by 3.5% (0.7–6.0%) [8].

The difference in analyzed patients between the operating room and PACU can be explained by the fact that, in some rare cases, patients were not admitted to the PACU postoperatively.

Besides the well-known limitations of conducting a retrospective analysis, the examiner is a factor that should not be neglected; the assessment of neuromuscular blockade in the tactile NMM group varies depending on the examiner. Due to the study’s retrospective design and changes of in-house standards, the data acquisition for the compared groups did not take place at the same time, as until August 2019 (in abdominal surgery) and December 2019 (in gynecological surgery), respectively tactile monitoring was the only available device and was replaced with quantitative NMM afterwards. We only included cases in which NMM was used, so no confounders for choosing one of the techniques can be analyzed.

When both arms are attached to the patients flank the acceleromyograph must be attached to the patient’s hand before positioning. With tactile monitoring the examiner had to touch the patients’ thumb which might make the examination more complicated and unhandy. It is not documented weather both arms were attached. It is also not documented whether all users of the quantitative NMM have performed a baseline evaluation before neuromuscular blockade, which is a potential bias. The factor of whether patients with a higher BMI were dosed according to their ideal body weight or their actual bodyweight was also not considered. The surgeons did not evaluate the surgical conditions. Preoperatively established peridural anesthesia may reduce the need for muscle relaxants. This was not investigated in the present study; further investigations should investigate this aspect in detail. Furthermore, no neuromuscular monitoring was performed in the PACU, therefore differences in oxygen demand between groups cannot be attributed to residual neuromuscular blockade.

## Conclusion

Rocuronium dosage given by the provider differs significantly depending on the monitoring system used. The significantly lower dosage, less repetitive applications, no significant difference in the need for antagonization and fewer respiratory complications suggest that using quantitative monitoring allows a more continuous neuromuscular blockade. Especially in laparoscopic surgery, patients might benefit from continuous neuromuscular blockade.
